# Supplementary material for: Identification and functional analysis of non-coding regulatory small RNA FenSr3 in Bacillus amyloliquefaciens LPB-18
Source: PeerJ. 2023 May 15;11:e15236. doi: 10.7717/peerj.15236 (PMC10194069; doi:10.7717/peerj.15236)
Supplement: Supplemental Information 4 [file peerj-11-15236-s004.zip › KO/CK-vs-T1_map/map05231.html]

KEGG PATHWAY: Choline metabolism in cancer - Reference pathway


|  |  |
| --- | --- |
| **Choline metabolism in cancer - Reference pathway** |  |

[
Pathway menu
| Organism menu
| Pathway entry
| Show description
| User data mapping
]

Abnormal choline metabolism is emerging as a metabolic hallmark that is associated with oncogenesis and tumour progression. Following transformation, oncogenic signalling via pathways such as the RAS and PI3K-AKT pathways, and transcription factors associated with oncogenesis such as hypoxia-inducible factor 1 (HIF1) mediate overexpression and activation of choline cycle enzymes, which causes increased levels of choline-containing precursors and breakdown products of membrane phospholipids. These products of choline phospholipid metabolism, such as phosphocholine (PCho), diacylglycerol (DAG) and phosphatidic acid, may function as second messengers that are essential for the mitogenic activity of growth factors, particularly in the activation of the ras-raf-1-MAPK cascade and protein kinase C pathway.

|  |  |  |
| --- | --- | --- |
| Reference pathway | 184% 150% 122% 100% 82% 67% 55% | 图片下载 |
